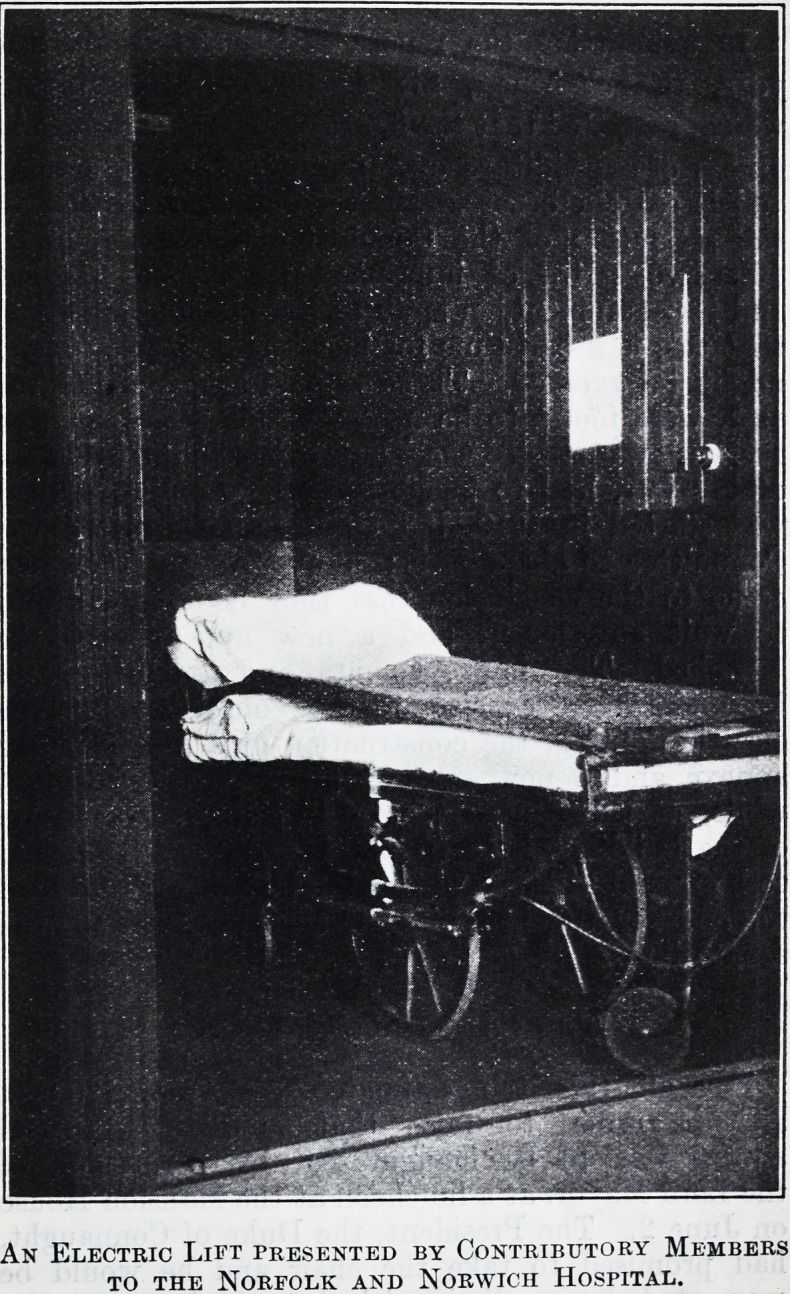# Contributory Benefactors

**Published:** 1924-06

**Authors:** 


### CONTRIBUTORY BENEFACTORS.

For many years two electric lifts were urgently needed at the Norfolk and Norwich Hospital, and eventually the Chairman and members of the Board of Management agreed to present one of these lifts to the Hospital at a cost of £1,250. It is the custom of the Hospital to invite representatives of the Contributory Scheme to visit the institution

An Electric Lift presented by Contributory Members to the Norfolk and Norwich Hospital.

on one Saturday afternoon each year, and at the following gathering of these representatives this offer of the Board was announced. One of the leading members of the Contributory Scheme Committee then moved that they should make a special effort to present the other lift. The suggestion was adopted and the sum of £1,250 was paid to the Hospital Authorities. The lift, of which we reproduce a photograph, is now installed.

### Additions to Inwood Cottage Hospital.

Through the generosity of Mr. F. B. Summers two new wards are being added to the Inwood Cottage Hospital at Alton, Hants. Each ward will accommodate nine patients, with the necessary annexes. The wards are on the ground floor, and patients can be wheeled into the garden. Mr. Goodwyn Hall has given the additional ground required. Mr. Summers has also added a new theatre unit and a new sitting-room for the m ron, over which will be two private wards, a nurses' room, bathroom and lavatory. The floors of all the new buildings will be of rubber. Mr. B. D. Canallor is the architect.

## Figures and Tables

**Figure f1:**